# Combination of Evidence from Bibliometrics and Bioinformatics Analysis Identifies miR-21 as a Potential Therapeutical Target for Diabetes

**DOI:** 10.3390/metabo14080403

**Published:** 2024-07-25

**Authors:** Yiqing Chen, Xuan Ye, Xiao Zhang, Zilin Guo, Wei Chen, Zihan Pan, Zengjie Zhang, Bing Li, Hongyun Wang, Jianhua Yao

**Affiliations:** 1 Institute of Geriatrics (Shanghai University), Affiliated Nantong Hospital of Shanghai University (The Sixth People’s Hospital of Nantong) and Shanghai Engineering Research Center of Organ Repair, School of Life Science, Shanghai University, Nantong 226011, China; chenyiqing970331@163.com (Y.C.); yexuan102030@163.com (X.Y.); zx642216184@163.com (X.Z.); gzl010418@163.com (Z.G.); 2 School of Traditional Chinese Medicine, Shanghai University of Traditional Chinese Medicine, Shanghai 201203, China; 3 Department of Emergency, Tongji Hospital, Tongji University School of Medicine, Shanghai 200065, China; chenwei_17@126.com; 4 QianWeiChang College, Shanghai University, 333 Nan Chen Road, Shanghai 200444, China; a3284564382@163.com (Z.P.); zzj13661539894@163.com (Z.Z.); 5 Department of Ophthalmology, Tongji Hospital, Tongji University School of Medicine, Shanghai 200065, China; bing.li@tongji.edu.cn; 6 Department of Cardiology, Shanghai Tenth People’s Hospital, Tongji University School of Medicine, Shanghai 200072, China; 7 Department of Cardiology, Shigatse People’s Hospital Tibet China, Shigatse 857012, China

**Keywords:** diabetes, bibliometrics and bioinformatics analysis, miR-21, extracellular vesicles

## Abstract

Many microRNAs (miRNAs) have been identified as being involved in diabetes; however, the question of which ones may be the most promising therapeutical targets still needs more investigation. This study aims to understand the overall development tendency and identify a specific miRNA molecule to attenuate diabetes. We developed a combined analysis method based on bibliometrics and bioinformatics to visualize research institutions, authors, cited references, and keywords to identify a promising target for diabetes. Our data showed that diabetes-related miRNA is receiving continuously increasing attention, with a large number of publications, indicating that this is still a hot topic in diabetes research. Scientists from different institutions are collaborating closely in this field. miR-21, miR-146a, miR-155, and miR-34a are frequently mentioned as high-frequency keywords in the related references. Moreover, among all the above miRNAs, bioinformatics analysis further strengthens the argument that miR-21 is the top significantly upregulated molecule in diabetes patients and plays an important role in the pathogenesis of diabetes. Our study may provide a way to identify targets and promote the clinical translation of miRNA-related therapeutical strategies for diabetes, which could also indicate present and future directions for research in this area.

## 1. Introduction

Diabetes mellitus is a major contributor to global mortality and has become a challenging public health issue [1]. According to a recent study published in the Lancet, the global number of diabetes cases reached around 529 million in 2021, affecting approximately 6.1% of the world’s population. This prevalence is expected to increase to 1.31 billion people by 2050 due to changes in lifestyle and an aging population, placing a significant burden on healthcare systems worldwide [2,3]. Most patients with diabetes have poor blood glucose control, often complicated by diabetic retinopathy, diabetic nephropathy, cardiovascular disease, etc. [4,5,6]. 

In recent years, research in the field of diabetes and its complications has received extensive attention; however, the pathological mechanism of diabetes is complex and there are numerous related references. It is necessary to periodically evaluate the research progress and trends in this field to provide references for early prevention and intervention strategies for diabetes and its complications.

MicroRNAs (miRNAs) are a type of small non-coding RNA of 20–24 nucleotides in length. They regulate gene expression by degrading or inhibiting the translation of target genes and participate in several biological processes, such as cell proliferation, apoptosis, and differentiation [7,8,9]. In addition to animals, miRNAs have also been found to play a distinct role in plant development, participating in almost all the developmental processes of plants, from larvae to adults [10,11,12]. Growing evidence indicates that miRNAs have a crucial role in diabetes and its complications [13,14,15]. Anna Zampetak’s team found that miR-20b, miR-21, and miR-24 in the peripheral blood of diabetic patients were significantly downregulated [16]. Yuan Xiong et al. reported that miR-20b-5p was significantly increased in circulating extracellular vehicles (EVs) in diabetic patients and inhibited wound healing and angiogenesis by regulating Wnt9b/β-catenin signaling [17,18]. The widespread presence of miRNAs in tissues and circulation (encapsulated in EVs) makes them promising new diagnostic markers and therapeutic targets for diabetes mellitus.

Bibliometrics is a multidisciplinary field that involves the quantitative analysis of all knowledge carriers using mathematical and statistical methods [19]. In recent years, diabetes research has become a major focal point. However, there is a lack of specialized bibliometric tools for the systematic analysis of the current state and trends of miRNAs related to diabetes [20]. 

We constructed a visualization map using CiteSpace 6.2.R4 and VOSviewer 1.6.18 software based on a bibliometric analysis of the literature. The map displays yearly publication volumes, author affiliations, research institutions, and keywords to demonstrate the current state, growth patterns, and research hotspots of miRNAs in diabetes. In addition, we analyzed publicly available sequencing data from the GEO database to uncover significant differences in miRNA expression between diabetic patients and those with complications. These findings provide valuable insights for future research in this area.

## 2. Materials and Methods

### 2.1. Literature Collection and Analysis

Considering the timeliness of the literature, we extracted the literature published between 31st August, 2013 and 31st August, 2023 from the Science Citation Index Expanded of the Web of Science Core Collection on a 10-year cycle. The search terms were TS = (“diabetes” or “diabetic”) AND TS = (“microRNA” or “miRNA”). A total of 4414 records were retrieved. According to the exclusion criteria for published bibliometric articles [21,22], we excluded 324 articles, including conference abstracts, book chapters, editorials, and other document types, and a total of 4088 articles and reviews were finally included.

**Figure 1:**
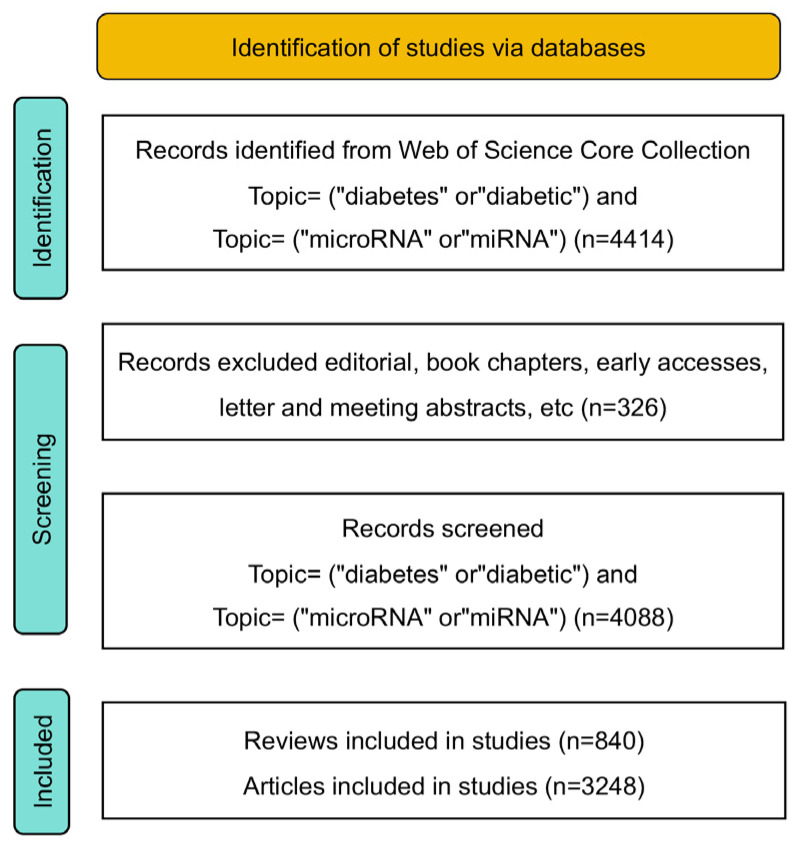
Flowchart of the literature selection. All data were collected from Web of Science Core Collection and underwent analysis with bibliometrics software CiteSpace 6.2.R4 and VOSviewer 1.6.18.

CiteSpace 6.2.R4 and VOSviewer 1.6.18 [23,24] were used to comb and visualize the data of the included 4088 valid citations in our prior study [25]. The relevant parameters were set as follows. The schedule slices were selected as 1 year per slice, and the time series was set from 2013 to 2023. By selecting institutions, authors, and keywords, the visualization analysis included timeline graphs of annual publication volume, a collaboration network of research institutions or authors, and high-frequency keyword clustering. Finally, we drew the PRISMA chart based on a template [26] to depict the literature selection process (Figure 1).

### 2.2. Microarray Data Collection and Analysis

Publicly available sequencing data for myocardial tissue samples (GSE44179) from diabetic cardiomyopathy rats and kidney tissue samples (GSE51674) [27] and skin trauma tissue samples (GSE188783) from diabetic patients and healthy individuals were collected from the GEO database on the NCBI platform. The online tool GEO2R (http://www.ncbi.nlm.nih.gov/geo/geo2r, accessed on 1 September 2023) was used to analyze the data and identify differential miRNAs, and ultimately draw the volcano plot graph with adjusted *p*-value < 0.05 and logFC ≤ −1 or logFC ≥ 1 [28].

### 2.3. Animals and Treatments

Adult male 6-week-old C57BL/6J mice were obtained from Charles River Laboratories (Shanghai, China). The mice were housed in a controlled environment at a temperature of 22 °C and with a 12/12-h light/dark cycle. To induce diabetic cardiomyopathy (DCM), the mice were fed a high-fat chow diet (Research diet, D12492) continuously. After 6 weeks, the mice received an intraperitoneal injection of streptozotocin (Sigma, Baltimore, MD, USA, #S0130, 20 mg/kg in 0.1 mmol/L sodium citrate buffer, pH 4.5) for 5 consecutive days. Control mice were injected with the same volume of sodium citrate buffer. Subsequently, the mice continued to be fed the high-fat diet for 12 weeks.

### 2.4. RNA Isolation and Relative Quantitative Real-Time PCR (Q-PCR)

Total RNA was isolated using the Trizol RNA extraction kit (TAKARA, Tyoko, tyo, Japan, 9109). Reverse transcription of 400 ng RNA was performed using iScript reverse transcription supermix (Bio-Rad, Hercules, CA, USA, 1708840) following the manufacturer’s instructions. Q-PCR analysis was conducted using SYBR Green (Bio-Rad, 1708880) on a LightCycler 480 Real-Time PCR System (Roche, Basel, Switzerland, 900067) with 5 s as control. miR-21 and 5 s primers were purchased from RiboBio Co., Ltd. (Guangzhou, China). The relative expression level was calculated using the 2^−ΔΔCT^ method.

### 2.5. Statistical Analysis

All data were expressed as mean ± SD. Significant differences between the two groups were calculated by independent-sample *t*-test. A *p*-value less than 0.05 was statistically different. The data were analyzed and graphed by using GraphPad Prism 8.0.

## 3. Results

### 3.1. Annual Trend of Publications in the Diabetes-Associated miRNA Field 

**Figure 2:**
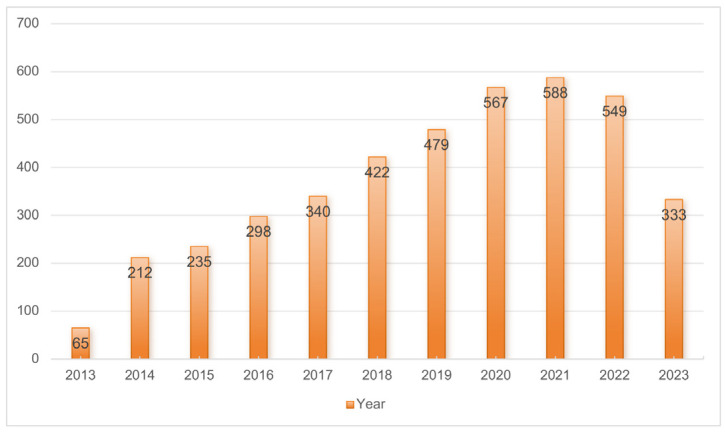
Trends of miRNA in the diabetic field published between 31 August 2013 and 31 August 2023.

To explore the research progress, trends, and frontiers of miRNAs in the field of diabetes mellitus, we used the bibliometric software CiteSpace and VOSviewer to visualize and analyze the annual publication volume of the relevant literature published in the past decade. As shown in Figure 2, there were only 65 related publications in 2013, followed by a sharp increase in the number of related publications from 2013 to 2021, which peaked in 2021 and then slightly declined in 2022. An annual average of 375.5 publications from 2013 to 2022 indicated that miRNA research was in a state of sustained development, which is still a hot direction in the field of diabetes research at present.

### 3.2. Distribution of Institutions in the Diabetes-Associated miRNA Field

**Figure 3:**
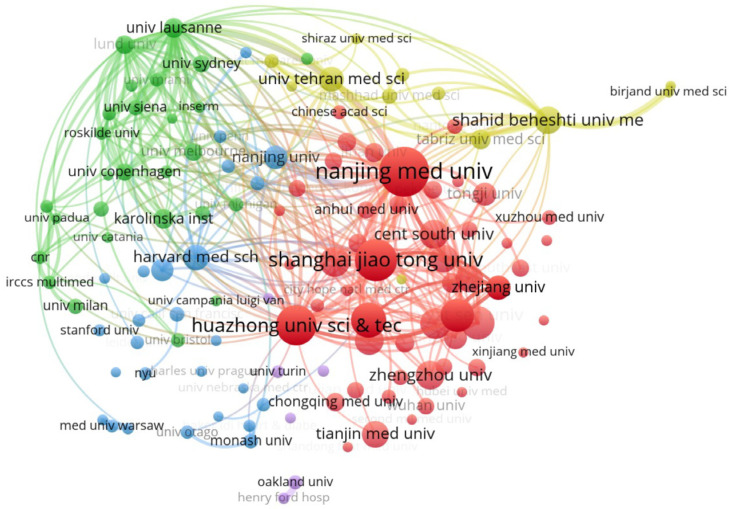
Institutional cooperation network of miRNA-and-diabetes-related studies. The size of the nodes in the map is directly related to the number of published authors and co-cited authors. The thickness of the lines is directly related to the strength of cooperation between authors and co-cited authors.

A total of 3793 institutions published literature related to “miRNA and diabetes”, and we visualized the top 122 institutions in terms of the number of publications (Figure 3). Specifically, the top 12 institutions with their number of published studies are shown in Table 1. Nanjing Medical University (105 articles), Shanghai Jiao Tong University (83 articles), and Huazhong University of Science and Technology (77 articles) are the top three institutions in terms of publication number, accounting for 2.6%, 2.0%, and 1.9% of the total number of articles, respectively. In addition, we can see an extensive collaboration between institutions based on the institutional relationship network diagram, and particularly strong collaborations between Nanjing Medical University and Nanjing University, Shanghai Jiao Tong University, and Harvard Medical School. The research system of these universities is relatively mature, and is closely related to the incidence of diabetes, the rate of patient visits, and the level of scientific research in the region. Establishing cooperative relationships and exchanges with them is conducive to removing academic barriers and further developing research related to diabetes and miRNAs.

### 3.3. Authors and Co-Cited Authors in the Diabetes-Associated miRNA Field

**Figure 4:**
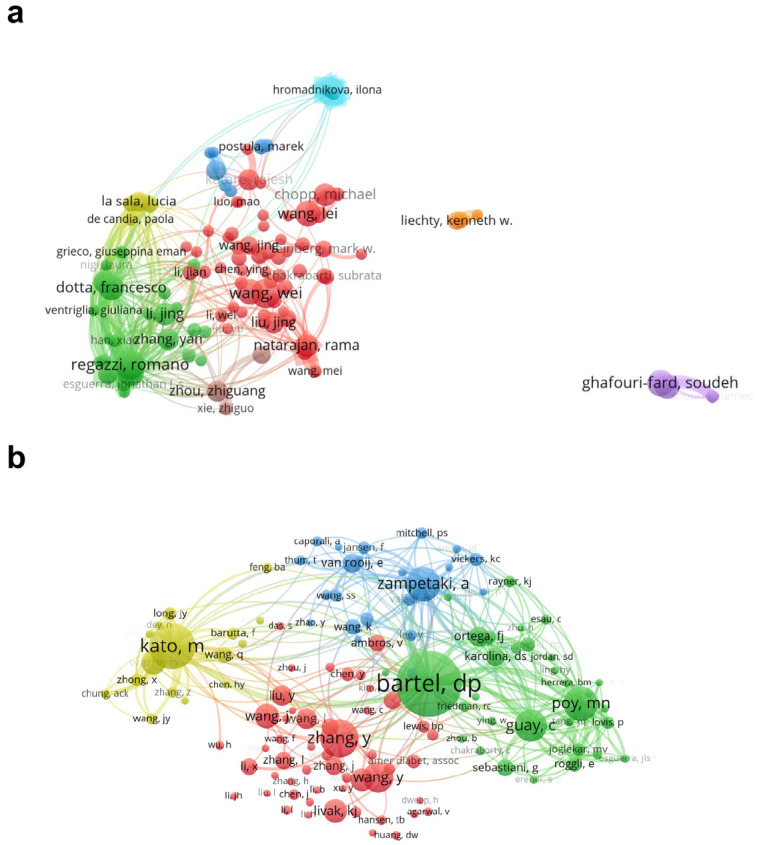
Visualization map of authors and co-cited authors involved in the miRNA and diabetes field. (**a**) The cooperative network of authors in the diabetes-associated miRNA field; (**b**) the cooperative network of co-cited authors in the diabetes-associated miRNA field. The size of the nodes in the map is directly related to the number of published authors and co-cited authors. The thickness of the lines is directly related to the strength of cooperation between authors and co-cited authors.

In the field of miRNAs and diabetes, a total of 20,846 authors have published related literature. We created a visualization network with the top 104 authors based on the number of publications (Figure 4a). The authors with the highest number of publications are Regazzi Romano from Lausanne University (21 publications), Wang Wei from Sun Yat-Sen University (21 publications), and Eliasson Lena from Lund University (19 publications). It is worth noting that Regazzi Romano has closely collaborated with Dotta Francesco. Furthermore, after analyzing the citation rankings and communication networks of co-cited authors (those cited at the same time by two or more authors), we found that there are 90,174 co-cited authors. Among them, three authors have been cited more than 500 times: Bartel David P, Kato Mitsuo, and Zhang Y (Figure 4b and Table 2). 

### 3.4. Keywords in the Diabetes-Associated miRNA Field

**Figure 5:**
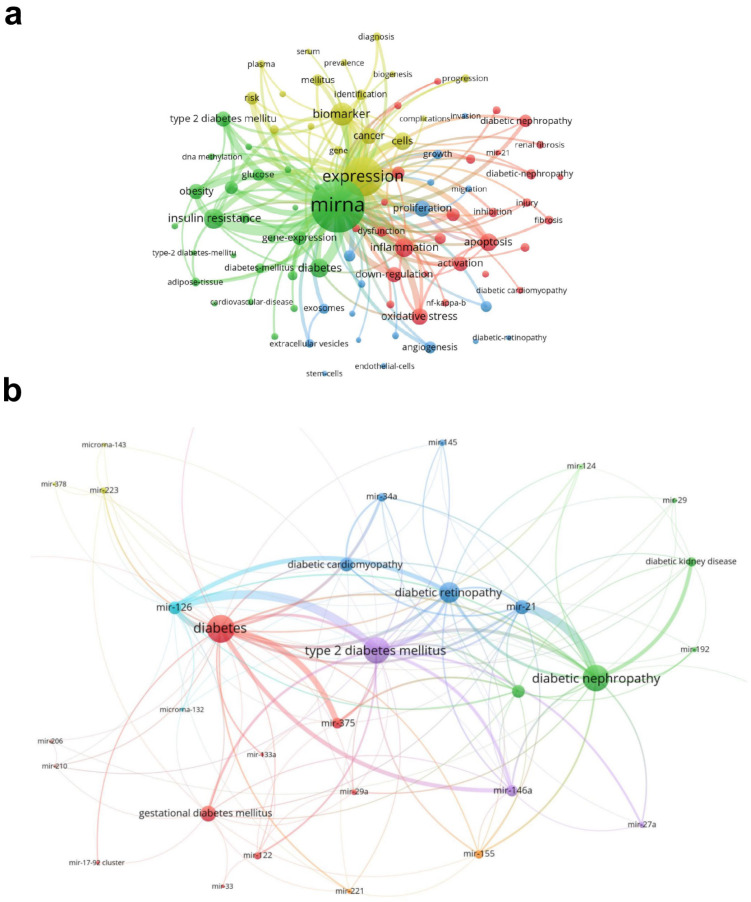
Visualization map of keyword clustering analysis on miRNA in diabetes. (**a**) A total of 11,885 keywords were included in the literature related to the miRNA and diabetes field. The visualization map of keyword co-occurrence was drawn with the top 90 keywords with the highest frequency. Four colors represented four different clusters. (**b**) The visualization map of keyword co-occurrence was drawn with keywords related to diabetes and miRNA family members. The size of the nodes in the map is directly related to the number of published authors and co-cited authors. The thickness of the lines is directly related to the strength of cooperation between authors and co-cited authors.

To further explore the current research status in the field of miRNA and diabetes, we applied VOSviewer software to analyze the keyword co-occurrence in the relevant literature. As shown in Figure 5a, a total of 11,885 keywords were involved. Notably, the keywords “miRNA and diabetes” were strongly linked to insulin resistance, oxidative stress, biomarkers, inflammation, and apoptosis, suggesting that miRNAs may mainly mediate the development of diabetes through oxidative stress, inflammation, apoptosis, and other related mechanisms. In addition, miRNAs may serve as biomarkers for diabetes. Moreover, from the analysis of these keywords, the mechanism, diagnosis, and treatment of diabetes are the current research hotspots. miRNAs and their downstream targets have also become the focus of research as a treatment for diabetes.

To clarify the most popular miRNA family members in the field of diabetes, we selected keywords related to type 2 diabetes mellitus/diabetes/diabetic complications, as well as miRNA family members, to draw keyword co-occurrence maps. Our results showed that the top ten high-frequency terms were miR-21, miR-126, miR-375, miR-146a, miR-155, miR-34a, miR-122, miR-223, miR-145, and miR-221, indicating that these miRNAs play a key role in the pathogenesis of diabetes and its complications (Figure 5b). 

### 3.5. miR-21 Is a Potential Therapeutical Target for Diabetes

**Figure 6:**
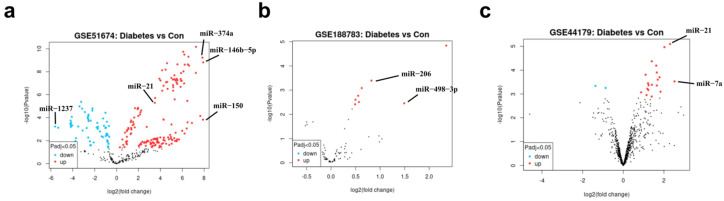
Identification of DEGs in the diabetes GEO dataset. (**a**) The volcanic map of differentially expressed miRNAs in kidney tissue samples from healthy controls and diabetic nephropathy patients; (**b**) the volcanic map of differentially expressed miRNAs in skin wound samples from healthy controls and diabetic patients; (**c**) the volcanic map of differentially expressed miRNAs in myocardial tissue samples from healthy controls and diabetic cardiomyopathy rats, with blue indicating downregulated miRNAs, red indicating upregulated miRNAs, and black indicating no statistical difference. Adjusted *p*-value < 0.05, logFC ≤ −1 or logFC ≥ 1 was considered as a different expression.

To further reveal the potential miRNA biomarkers and targets of diabetes and its complications, we obtained sequencing datasets of diabetes (GSE51674, GSE188783, and GSE44179) from the GEO database and applied the GEO2R online analysis tool to analyze and draw the volcano maps. Analysis of the kidney tissue sequencing dataset for diabetic nephropathy patients (GSE51674) and skin wound tissue sequencing dataset for diabetes mellitus patients (GSE188783) revealed 762 and 7 differentially expressed miRNAs, respectively. Among these, miR-146b-5p, miR-150, miR-146a, miR-21, etc., were significantly upregulated in kidney tissue, while miR-1237, miR-933, and others were downregulated (Figure 6a,b). In addition, 21 differentially expressed miRNAs were obtained by analyzing serum tissue microarray data from the myocardial tissue of diabetic cardiomyopathy rats (GSE44179), including 19 upregulated and 2 downregulated miRNAs (Figure 6c). Through a combination of bibliometrics and bioinformatics analysis, we found that miR-21 was the most significantly upregulated miRNA and had the highest frequency in the keyword analysis. These findings suggest that miR-21 may be a potential target for the treatment of diabetes and diabetic complications.

### 3.6. Hot Topics and Possible Directions in the Field of miRNAs and Diabetes

**Figure 7:**
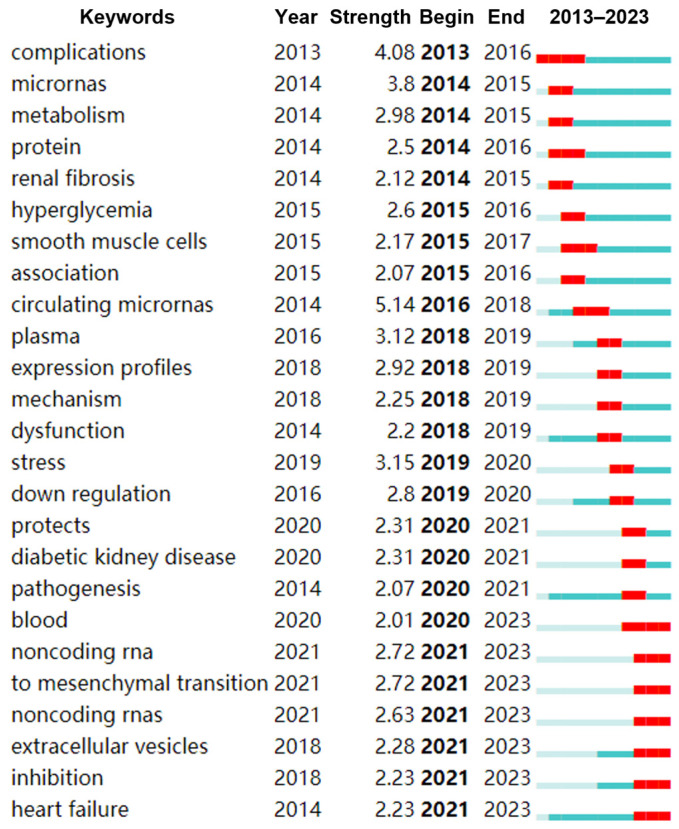
Top 25 keywords with the strongest citation bursts involved in miRNA and diabetes field. The red section indicates the period when the keyword with the strongest citation bursts, and the blue section indicates the period when the keyword with the less citation bursts.

To better understand the research trends in the field of miRNAs and diabetes, we analyzed emerging keywords and identified the top keywords with the strongest citation bursts. Our findings revealed the top 25 emerging keywords, with “non-coding RNA, mesenchymal transition, extracellular vesicles, inhibition, and heart failure” showing significant increases in citations between 2021 and 2023. This suggests that mesenchymal transition and extracellular vesicles may be prominent areas of research in the field of miRNAs and diabetes in recent years (Figure 7). 

**Figure 8:**
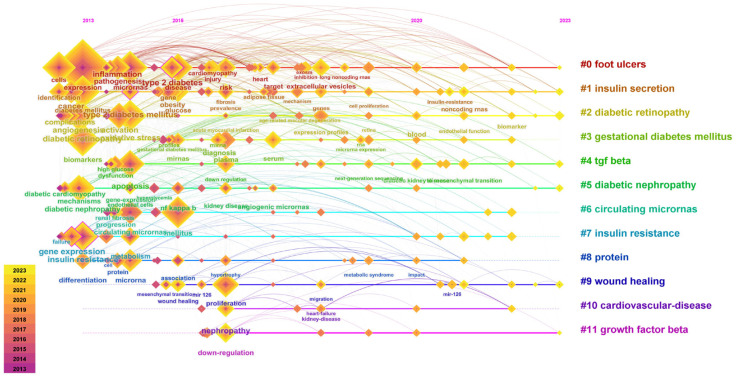
The visualization timeline map of miRNA and diabetes studies. The *x*-axis is the published year of keywords, and *y*-axis is the cluster number, which shows the period and research process of each cluster.

We then performed a timeline graph clustering analysis of keywords in the field of miRNAs and diabetes using the Citespace 6.2.R4 software to explore the period and research process of the 12 keyword clusters (Figure 8). Our findings revealed that the keyword clusters focused on various diabetic comorbidities, such as diabetic retinopathy, gestational diabetes, diabetic nephropathy, and cardiovascular disease. The related studies in clusters 0 (foot ulcer), 1 (insulin secretion), and 2 (diabetic retinopathy) spanned from 2013 to 2023 and focused on keywords such as pathogenesis, type 2 diabetes, injury, cancer, angiogenesis, and activation. The related studies in clusters 10 (cardiovascular disease) and 11 (growth factor beta) appeared most recently, with the keywords mainly including microRNA signature and small RNA. The keyword clustering timeline graph indicated that miRNA research in the field of diabetes is relatively mature; however, the interest and attention from scientific researchers and medical professionals remain constant.

### 3.7. Small Molecules with Inhibitory Effects on miR-21

**Figure 9:**
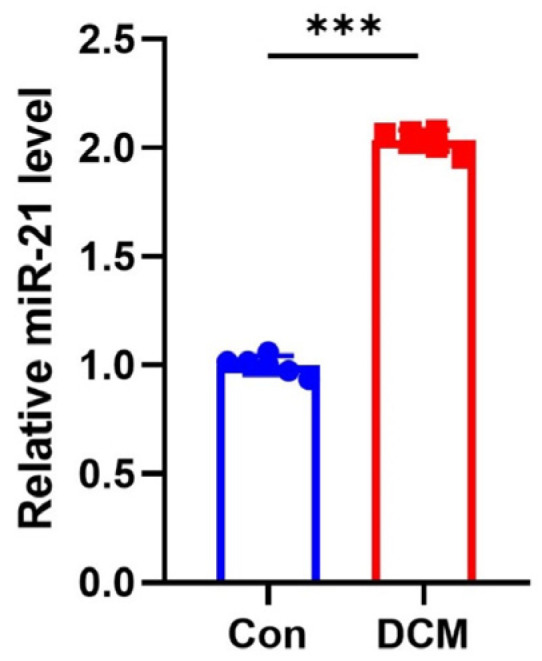
Q-PCR showed the expression change of miRNA in DCM mouse heart tissue. N = 6. *** *p* < 0.001 versus respective control.

Through combined bibliometric and bioinformatics analysis, we deduced that miR-21 may be a key factor involved in the pathogenesis of diabetes and its complications. To further validate our conclusion, we used Q-PCR to detect the expression level of miR-21 in the cardiac tissues of DCM mice. Consistent with previous predictions, miR-21 was significantly upregulated in the hearts of mice with DCM (Figure 9).

It has been shown in numerous studies that bioactive small molecules, or drugs, can control the expression of miRNAs. This suggests that targeting miRNAs with small molecules could be a promising approach for treating human diseases. Since miR-21 is thought to be a key potential target for diabetes and its complications, we used the SM2miR database (http://www.jianglab.cn/SM2miR/, accessed on 1 September 2023) [29], a database that records small molecules and miRNAs, and detailed information about them, to search for possible small-molecule drugs for diabetes. We found multiple small molecules that have inhibitory effects on miR-21, including Hydroxychloroquine, Prednisone, Trypaflavine, 5-fluorouracil, etc. (Table 3). These small molecules may become effective therapeutic drugs for diabetes and its complications. However, further experiments are necessary to validate these findings.

## 4. Discussion

In recent years, numerous studies have suggested that miRNAs play a role in various cell biological processes, affecting the progression of diabetes, and have been identified as potential biomarkers and targets for the clinical diagnosis and treatment of diabetes mellitus [30,31,32]. Despite the extensive literature in this field, there is a lack of systematic and comprehensive analysis. Therefore, we built a combination strategy based on bibliometrics and bioinformatics analysis and identified miR-21 as a possible therapeutical target for diabetes treatment. In addition, we found that EV-enclosed miRNAs may represent a promising direction for future diabetic studies. Our findings offer a novel strategy for identifying treatment targets and research directions for human diseases.

During the past decade, there has been a steady increase in miRNA-related research on diabetes and its complications. The total of 4088 relevant publications indicated a growing interest in this field, making it a current research focus. The source institutions of this literature are numerous and exist in close cooperation, among which Nanjing Medical University has published the most literature in this field. Furthermore, a total of 20,846 authors have published relevant literature in this field, among which the Regazzi Romano team has the most research results, with a total of 1222 citations. These data indicated that close cooperation and communication among authors, research teams, and institutions could promote the high-quality production of scientific studies.

To further identify potential targets for diabetes treatment, we built a combination strategy based on bibliometrics and bioinformatics analysis. We screened and analyzed the literature and drew a citation keyword burst map, which indicated that miR-21 appeared as the most high-frequency keyword in the field of miRNAs and diabetes. Moreover, we conducted bioinformatics analysis on publicly available sequencing data and found that miR-21 was the most upregulated molecule in diabetic patients. miR-21 may be a promising biomarker and a therapeutical target for diabetes [33,34]. 

In particular, the analysis of keyword citation bursts reveals a significant increase in the number of citations related to miRNA-carrying extracellular vesicles in recent years [35]. As an important mediator of intercellular communication, EVs can carry and deliver miRNAs and other important signaling molecules to distal cells and organs, ultimately leading to the development of physical or pathological processes [35,36,37]. At present, a variety of studies have identified EV-enclosed miRNAs involved in regulating the development of diabetes [38,39]. For example, Wei Ying et al. found that miR-690 is highly expressed in M2-polarized bone marrow-derived macrophage exosomes and is involved in the regulation of insulin signal transduction [40]. Yi-Chun Tsai’s team found that a hyperglycemic environment stimulated renal proximal tubule secretion of EVs carrying miR-92a-5p, leading to glomerular plasma cell injury [41]. These findings demonstrate that EVs currently represent a hotspot and a new direction for miRNA research in the field of diabetes. 

This study has some limitations that need further investigation. We only analyzed the literature included in the Web of Science Core Collection and excluded books, conference papers, etc., which could have led to possible one-sidedness in the analysis results. Meanwhile, the bibliometrics-analyzing software may have some inaccurate identification of some keywords. Specifically, bibliometrics is a method of quantitatively analyzing a large amount of the literature through statistical, mathematical, and computer science methods, and the quantitative indicators of this method focus on superficial indicators such as citation counts and impact factors, which, when combined with bioinformatics, may result in the neglect of some important but less cited findings. In addition, the rapid development of bioinformatics and the continuous innovation in various sequencing technologies, given that bibliometric analyses have poor timeliness and the results are mostly based on the validation of research reports, may result in the lack of timely feedback.

In summary, the limitations of combining bibliometrics and bioinformatics are mainly reflected in the defects of bibliometric methods and the challenges brought by the rapid development of bioinformatics. In the future, with the continuous progress of technology, the combination of these two research methods needs to be constantly innovatively developed and improved to better serve academic research and development.

## 5. Conclusions

In summary, we built a strategy based on a combination of bibliometrics and bioinformatics analysis and identified that miR-21 could be a promising target for diabetes treatment, which may promote the development of molecular drugs. In addition, we systematically sorted out the research lineage of diabetes-related miRNAs and visualized the annual trends in research, the distribution of institutions and authors, and the research hotspots and hot topics in the field of miRNAs and diabetes. EV-enclosed miRNAs may represent a promising research approach in treating diabetes and its complications.

## Figures and Tables

**Table 1:** Distribution of publications from institutions about miRNA in the field of diabetes.

No.	Institution	Documents	Citations	Average Citation
1	Nanjing Medical University	105	3746	35.68
2	Shanghai Jiao Tong University	83	2130	25.66
3	Huazhong Univ Sci and Technol	77	1485	19.29
4	Harbin Medical University	64	1533	23.95
5	Fudan University	61	2369	38.84
6	Sun Yat-Sen University	58	1609	27.74
7	Southern med University	55	1510	27.45
8	China Medical University	53	1030	19.43
9	Capital Medical University	51	1099	21.55
10	Cent South University	50	828	16.56

**Table 2:** Top 12 authors and co-cited authors related to miRNA in the field of diabetes.

No.	Author	Documents	Citations	Total Link Strength	No.	Co-Cited Author	Citations	Total Link Strength
1	Regazzi, Romano	21	1222	18,409	1	Bartel, David P	921	7549
2	Wang, Wei	21	578	4122	2	Kato, Mitsuo	644	7005
3	Eliasson, Lena	19	708	15,544	3	Zhang, Y	535	4242
4	Ghafouri-fard, Soudeh	19	131	4844	4	Zampetaki, Anna	490	5580
5	Dotta, Francesco	18	725	13,981	5	Poy, Matthew N	486	5614
6	Li, Yang	18	442	2219	6	Guay, Claudiane	458	5073
7	Wang, Lei	18	548	5069	7	Wang, Y	427	3417
8	Taheri, Mohammad	17	116	4730	8	Wang, J	345	3119
9	Chopp, Michael	16	634	5005	9	Wang, B	343	4036
10	Li, Jing	16	447	4658	10	Livak, Kenneth J	336	1421
11	Natarajan, Rama	16	1224	9285	11	Li, Y	319	2250
12	Sebastiani, Guido	16	684	11,995	12	Wang, L	290	2515

**Table 3:** List of small molecules that have inhibitory effects on miR-21.

No.	Small Molecule	DrugbankID	CID
1	Hydroxychloroquine	DB01611	3652
2	Prednisone	DB00635	5865
3	Trypaflavine	DB03843	712
4	5-fluorouracil	DB00544	3385
5	Gemcitabine	DB00441	60750
6	5-aza-2′-deoxycytidine (5-Aza-CdR)	DB01262	451668
7	Sulindac sulfide	DB00605	5352624
8	17beta-estradiol (E2)	DB00783	5757
9	Sevoflurane	DB01236	5206
10	Nicotine	DB00184	89594
11	Morphine	DB00295	5288826

## Data Availability

All data generated or analyzed during this study are included in this article.
